# Computational Investigations on the Effects of Gurney Flap on Airfoil Aerodynamics

**DOI:** 10.1155/2015/402358

**Published:** 2015-01-14

**Authors:** Shubham Jain, Nekkanti Sitaram, Sriram Krishnaswamy

**Affiliations:** ^1^Department of Aerospace Engineering, PEC University of Technology, Sector 12, Chandigarh 160012, India; ^2^Department of Mechanical Engineering, IIT Madras, India; ^3^Department of Mechanical Engineering, BITS-Pilani, Hyderabad Campus, Jawahar Nagar, Shameerpet Mandal, Hyderabad, Telangana 500078, India

## Abstract

The present study comprises steady state, two-dimensional computational investigations performed on NACA 0012 airfoil to analyze the effect of Gurney flap (GF) on airfoil aerodynamics using *k*-*ε* RNG turbulence model of FLUENT. Airfoil with GF is analyzed for six different heights from 0.5% to 4% of the chord length, seven positions from 0% to 20% of the chord length from the trailing edge, and seven mounting angles from 30° to 120° with the chord. Computed values of lift and drag coefficients with angle of attack are compared with experimental values and good agreement is found at low angles of attack. In addition static pressure distribution on the airfoil surface and pathlines and turbulence intensities near the trailing edge are present. From the computational investigation, it is recommended that Gurney flaps with a height of 1.5% chord be installed perpendicular to chord and as close to the trailing edge as possible to obtain maximum lift enhancement with minimum drag penalty.

## 1. Introduction

### 1.1. Gurney Flap

**Figure 1:**
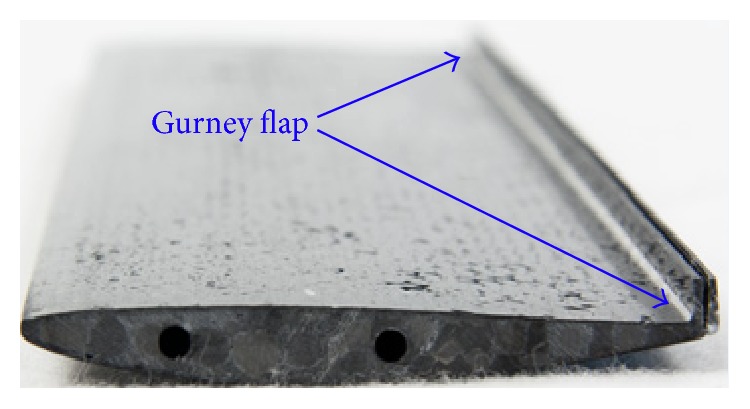
GF on wing trailing edge.

A Gurney flap (GF) is a microtab fitted to the airfoil near the trailing edge on its pressure side as shown in Figure 1. It was first used by Dan Gurney on the top trailing edge of the rear wing on his race car to provide extra rear-end down force with minimal aerodynamics disturbance [1]. Liebeck [2] conducted first wind tunnel experiments on GF. Over the decades, Gurney flap has attracted the attention of engineers and designers by its performance enhancement. A Gurney flap is easy to analyze and manufacture because of its very simple design. The Gurney flaps are extensively used on helicopters such as Apache AH-64, Sikorsky S76, and Eurocopter AS355 [3] and much research is going on for their use in turbomachines [4]. An excellent review of GF research for aircraft wings and other aerodynamics applications was presented by Wang et al. [5].

Jang et al. [6], Yoo [7], and Li et al. [8] have verified the lift enhancement of GF in their experiments. Neuhart and Pendergraft [9] visualized recirculation zones behind GF in their water tunnel experiments and also recommended to keep the GF height less than 2% of the chord length to reduce drag penalty which was also verified by Myose et al. [10]. Experiments on GF for GU25-5(11)-8 airfoil by Galbraith [11] concluded that GF should be mounted at distance *S* < 10% to prevent major performance degradation as verified by Li et al. [12] for NACA 0012 airfoil.

Brown and Filippone [13] conducted experiments at Reynolds number ranging from Re = 42000 to 1.6 × 10^5^. Their analysis also shows that the optimum height of GFs is always below the boundary layer thickness at the trailing edge. Nonlinear relation in lift increment and GF height was also noticed, where lift increased for small GFs rapidly while rate was slower for big flaps. Saturation in lift for larger GFs is also suggested by Hage et al. [14]. Hysteresis effect is observed by Brown and Filippone [13], when the flow reattachment takes place at lower AoA when incidence is decreased from poststall angles.

The earlier numerical investigations by Fripp and Hopkins [15], Myose et al. [16], and Jeffrey [17], who used panel methods to model sections fitted with GFs, reported disappointing results following comparison with experimental data.

RANS investigations have been carried out recently by Date and Turnock [18], Lee and Kroo [19, 20], Tongchitpakdee et al. [21], Li and Shen [22], and Singh et al. [23] with various parameters of the GF being systematically investigated. Good comparison with the experimental studies is obtained as long as sufficiently fine grids had been used and the correct time step is used for the time accurate simulations.

Chen et al. [24] computationally investigated the effects of square, round, and smooth convex configurations of the GF in low-solidity low-pressure turbine cascade and found the round configuration to be most effective to decreasing the adverse pressure gradient by increasing the flow turning angle and reducing the flow losses in low-solidity cascade. T strip is found to increase the slope of lift curve without any shift in zero lift angle by Cavanaugh et al. [25].

Considerable experimental and computational efforts are carried out on the effects of GF on airfoil aerodynamics. However, there are no systematic investigations on the effect of various parameters of GF (Reynolds number, height, position, mounting angle, configuration, etc.) on airfoil aerodynamics. Hence the present investigation is undertaken with the main aim of investigating computationally the influence of these parameters on the airfoil performance in a systematic manner. GF was analyzed for six different heights ranging from 0.5% to 4% at the trailing edge perpendicular to the chord. Trailing edge of the wing is generally thin and may not be able to support the flap due to structural factors. Hence, GF with *H* = 1.5% is investigated near the trailing edge for seven different positions from 0% to 20% mounted perpendicularly to the chord (Table 2). Effect of mounting angle is studied with GF of *H* = 1.5% mounted at seven different values of Φ from 30° to 120° with the chord to cover a wide range of mounting angles.

## 2. Geometry and Grid Generation

**Figure 2:**
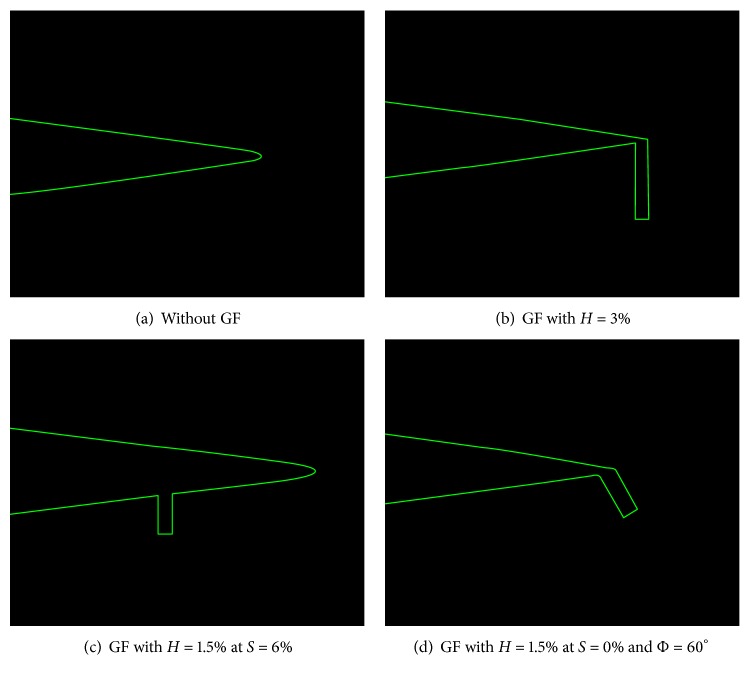
Trailing edge of airfoil with various GF geometries.

Airfoil considered in this study is NACA 0012 airfoil with chord length of 1 m. C-type domain and grid are created in ICEM CFD with far-field boundaries 12.5 chords away from trailing edge in all directions.  Figure 2 presents closeup of trailing edge of NACA 0012 airfoil with various GF geometries created in ICEM CFD.

**Figure 3:**
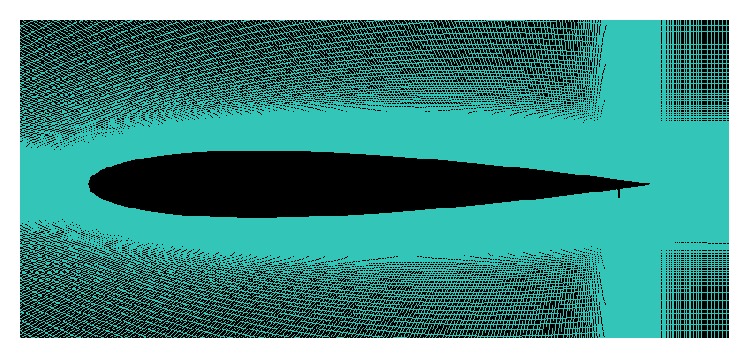
Fine layer of grid cells around the airfoil boundary generated in ICEM CFD.

For GF investigation, the grids are generated in ICEM CFD with at least 200,000 nodes as verified by grid dependency studies done by Krishnaswamy et al. [26]. Flow around the airfoil with GF is highly complex with high intensity vortices. Hence, a very fine grid layer with grid cell width of 0.5 mm is generated around the airfoil as shown in Figure 3.

## 3. Computational Methodology

### 3.1. Turbulence Modeling

For GF investigation at high Reynolds number *k*-*ε* RNG model is chosen as verified by turbulence model dependency studies by Krishnaswamy et al. [26].

### 3.2. Governing Equations

Flow field for all the simulations is assumed to be fully turbulent. As for all the cases, Mach number is always less than 0.3, flow is incompressible [27], and hence the energy equation is not used for numerical simulations.

*For k-ε RNG Turbulence Model*. The turbulence kinetic energy, *k*, and its rate of dissipation, *ε*, in *k*-*ε* RNG turbulence model are obtained from the following transport equations [28]:

(1) ∂∂tρk+∂∂xiρkui  =∂∂xjαkμεζf∂k∂xj+Gk+Gb+ρε−YM+Sk,∂∂tρε+∂∂xiρεui  =∂∂xjαεμεζf∂ε∂xj+C1εεkGk+C3εGb   −C2ερε2k−Rε+Sε.

In these equations, *G*_*k*_ represents the generation of turbulence kinetic energy due to the mean velocity gradients, whereas *G*_*b*_ is the generation of the turbulent kinetic energy due to buoyancy. *Y*_*M*_ represents the contribution of fluctuating dilation in compressible turbulence to overall dissipation rate. *α*_*k*_ and *α*_*ε*_ are the inverse Prandtl numbers for *k* and *ε*, respectively. Values of model constants have been derived analytically by the RNG theory.

### 3.3. Boundary Conditions and Solver Settings

The airfoil boundary is assigned as solid-wall with no-slip condition while inlet is assigned as velocity inlet and outlet is assigned as pressure-outlet conditions. Density based implicit solving scheme is used with the flow medium being air and Mach number less than 0.3. Hence the fluid is assumed to be incompressible with constant density of 1.225 kg/m^3^ and dynamics viscosity of 1.7894 × 10^−5^ kg/m-s. The value of Reynolds number based on chord and inlet velocity is 2.1 × 10^6^ equal to that of experimental investigations [8, 12].

First order upwind is used for calculating the transport variables for each turbulence model. Under relaxation factors for all the transport variables are set to 0.8. Solution initialization is computed from velocity inlet followed by FMG initialization with solution steering. Equations are solved until a convergence criterion of 10^−5^ for all the residuals is satisfied.

## 4. Effect of Height of GF on Airfoil Aerodynamics

Results obtained from CFD are compared with the available experimental results from Li et al. [8, 12].

### 4.1. Lift Coefficient

**Figure 4:**
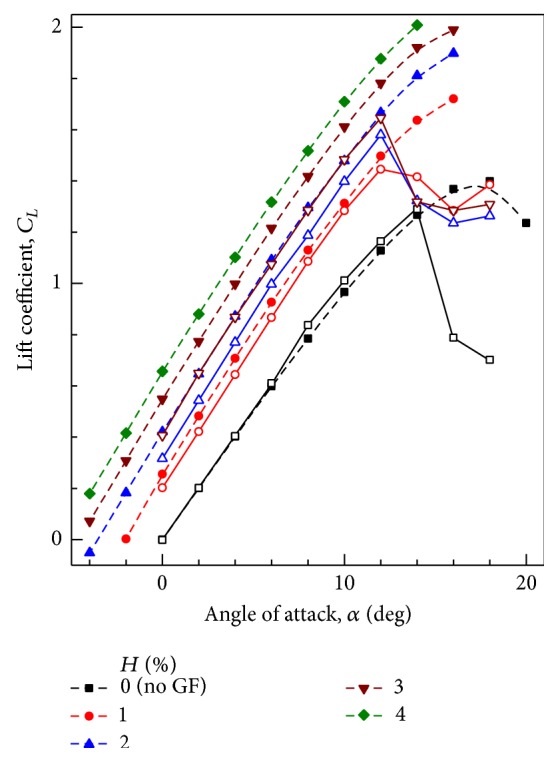
Variation of lift coefficient with angle of attack at different GF heights. Closed symbol + dashed line: Computational results. Open symbol + solid line: Experimental results [8].

**Figure 5:**
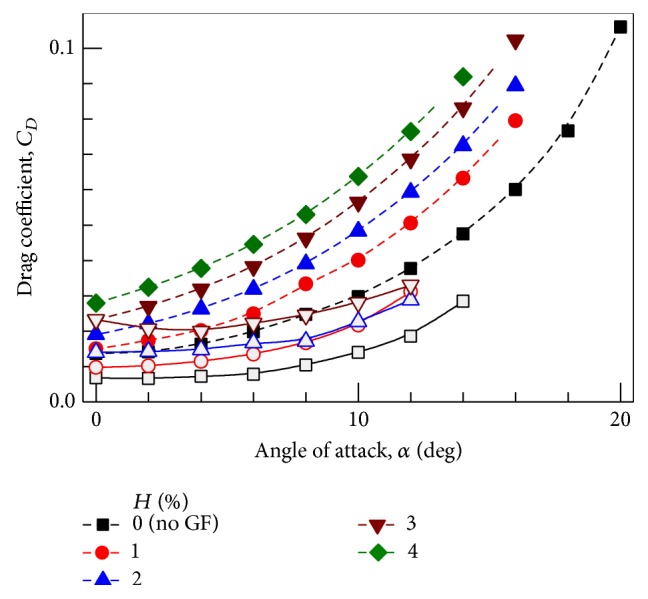
Variation of drag coefficient with angle of attack at different GF heights. Closed symbol + dashed line: Computational results. Open symbol + solid line: Experimental results [8].

The variation of lift coefficient with AoA is presented in Figures 4 and 5. The values of *C*_*L*_ with GF heights of 0% (without GF), 1%, 2%, 3%, and 4% are presented. The computed values of lift coefficient agree well with the experimental results up to the stall angle. At the stall angle, the experimental value of lift coefficient drops abruptly, while the computed lift coefficient continues to increase. Krishnaswamy et al. [26] used different turbulence models available in the commercial software FLUENT. Some of the models did not converge near and above stall angle. Computations done with *k*-*ε* RNG turbulence model provided converged solutions for AoAs near and above stall angle. Hence these results are included. An improved turbulence model may provide more accurate results near and above stall angle. When compared with clean airfoil at a given AoA of 10°, increase in *C*_*L*_ for 0.5%, 1%, 1.5%, 2%, 3%, and 4% flap height is 25%, 36%, 47%, 53%, 67%, and 77%, respectively. Apart from increasing the *C*_*L*_ values at a given incidence, maximum *C*_*L*_ values compared with clean airfoil are also increased by 19%, 23%, 31%, 36%, 42%, and 44% when flap heights of 0.5%, 1%, 1.5%, 2%, 3%, and 4% are used respectively. The increment in maximum *C*_*L*_ decreases as the flap height increases.  Table 1 compares the *C*_*L*_ values at AoA = 12° for airfoil with different GF heights.

### 4.2. Drag Coefficient

The variation of drag coefficient with AoA is presented in Figure 5.* L/D* ratio increases up to 2% GF height, but the drag overpowers the* L/D* ratio if the flap height is increased further. Drastic rise in drag for 3% and 4% flap heights, which results in lower* L/D* ratio, is in accordance with experimental results. For low *C*_*L*_ values, drag penalty is associated but* L/D* ratio is higher for moderate *C*_*L*_ values. Also for a given* L/D* ratio, *C*_*L*_ values are higher for larger flap height.

Experimental drag values are nearly constant for initial AoAs and drastic rise in drag is shown near stall. But these trends are not correctly predicted computationally, where increment in *C*_*D*_ is almost continuous. From experimental results minimum *C*_*D*_ values with GF are found at positive but small AoA, signifying the increased camber of the originally symmetric airfoil which is not predicted in computational results.

### 4.3. Static Pressure on Blade Surfaces

**Figure 6:**
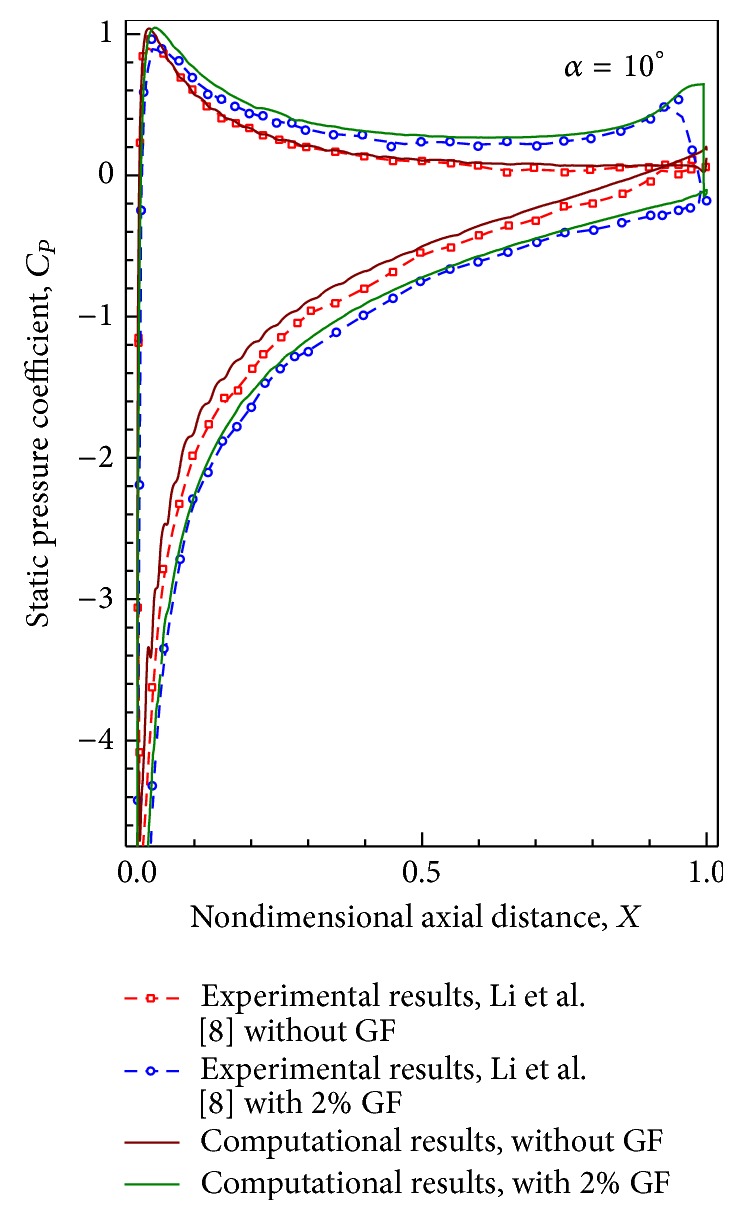
Static pressure distributions for different angles of attack for 2% GF.

Distribution of static pressure coefficient on the airfoil surface obtained experimentally and computationally with 2% height GF is compared for AoAs of 10° in Figure 6. For both airfoils without and with GF, the agreement between experimental and computed static pressure distribution is very good even near GF. Increased suction on suction surface and increased pressure on pressure surface are clearly noticeable on installation of GF, which results in lift enhancement.

**Figure 7:**
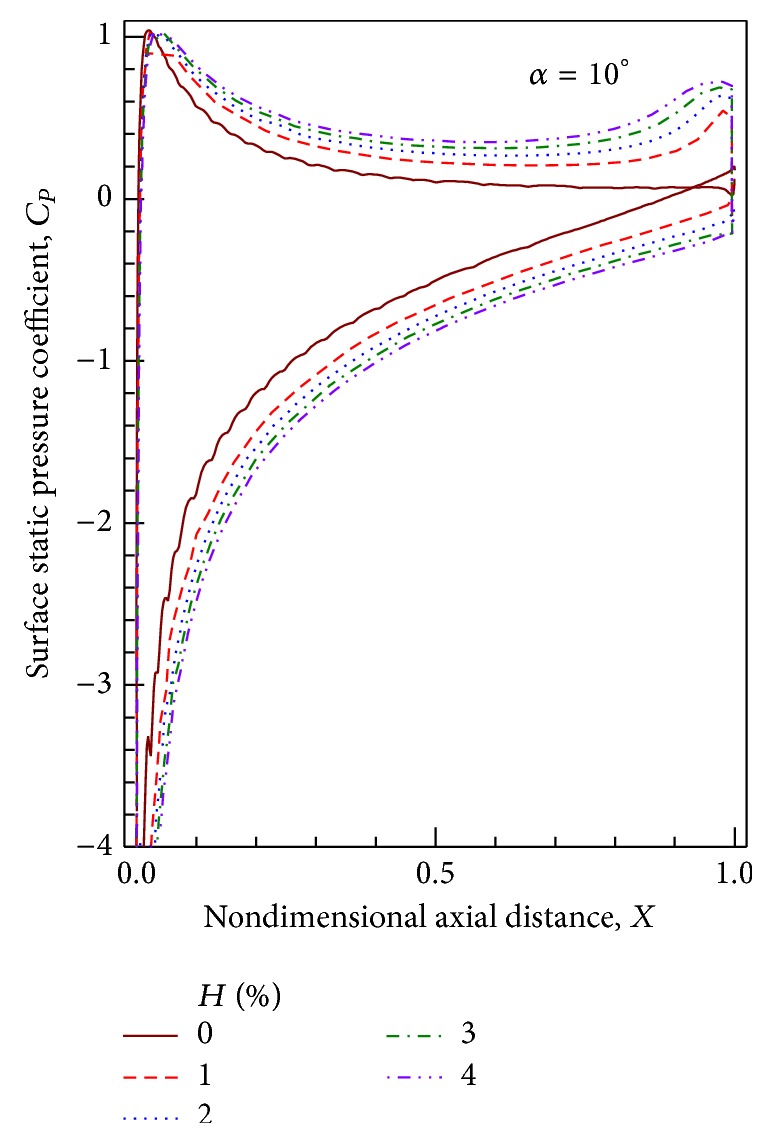
Static pressure distributions for different GF heights at AoA = 10°.

Static pressure distribution at an AoA = 10° for different GF heights is shown in Figure 7. Although suction is increased throughout the surface, the difference is maximum near the trailing edge where the GF is installed. When GF height is increased the maximum suction on the suction surface increases by 27.5%, 39.6%, 50.2%, and 60.3%, respectively, when the GF height is 1%, 2%, 3%, and 4% compared to that on the airfoil without GF. However the difference in static pressure distribution reduces as the GF height increases.

### 4.4. Pathlines and Turbulence Intensity

**Figure 8:**
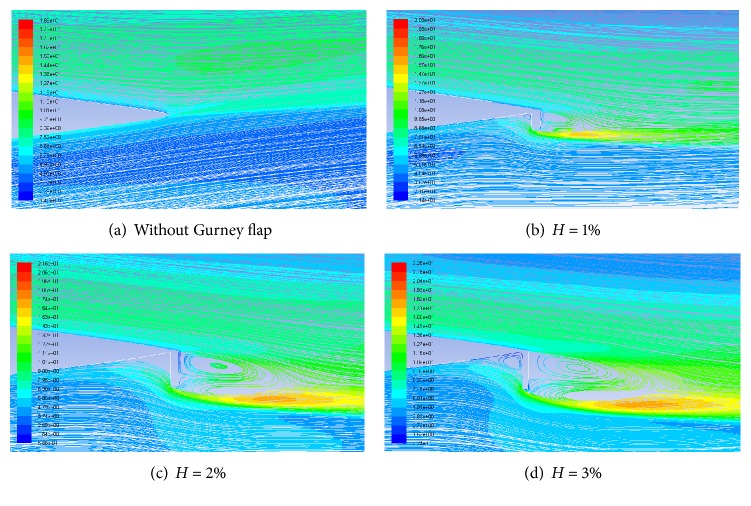
Pathlines and turbulence intensities for different GF heights at AoA = 10°.

Flow near the trailing edge of airfoil with and without GF at an AoA of 10° is compared in Figure 7 with the help of pathlines and turbulence intensity. Two counter rotating alternatively shed vortices with high turbulence intensity are clearly visible in the wake of the GF. In addition to vortices in the wake, one more vortex region is created in front of the flap. These vortices are responsible for the increased suction on suction surface and increased pressure on the pressure surface which is ultimately responsible for the enhanced lifting capability of the Gurney flap. As the height of Gurney flap increases, the strength of the vortex increases which results in more deflection of the flow at the trailing edge towards the flap, hence increasing the effective downwash (Figure 8).

## 5. Effect of Position of GF on Airfoil Aerodynamics

### 5.1. Lift Coefficient

**Figure 9:**
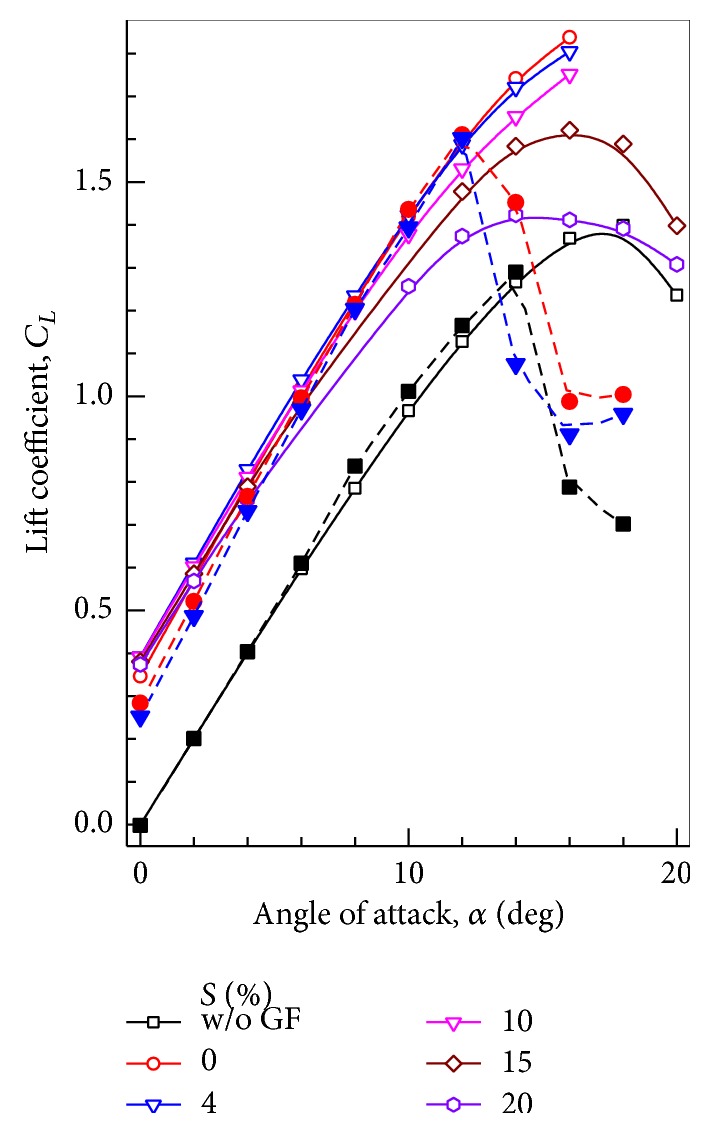
Variation of lift coefficient with angle of attack for GF positions. Open symbols + solid line: Computational results. Closed symbols + dashed line: Experimental results [12].

**Figure 10:**
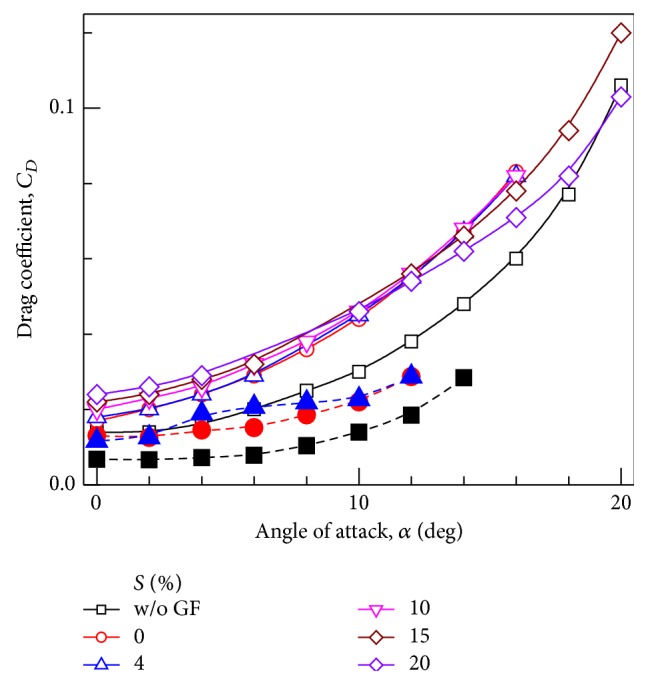
Variation of drag coefficient with angle of attack. Open symbols + solid line: Computational results. Closed symbols + dashed line: Experimental results [12].

Figures 9 and 10 compare experimental and computational values of *C*_*L*_ and *C*_*D*_ for different positions of GF. The height of GF is fixed at 1.5% of chord. This height is chosen for computational investigation of effects of position and mounting angle of GF as the experimental results [8] show that the drag coefficient remains nearly constant for *H* = 1 to 2% and increases rapidly beyond *H* = 2%. Further experimental results are available only for this height for various values of GF position and mounting angle [12]. Brown and Filippone [13] proposed the following semiempirical formula linking flap height to free stream velocity and airfoil chord length:

(2) $$\begin{array}{c} h_{\text{opt}}=37.155\left(\frac{{\text{Ch}}^{0.8}}{U^{0.2}}\right), \end{array}$$

where *h*_opt_ = optimum GF height (mm), Ch = airfoil chord (m), and *U* = free stream velocity (m/s).

From the above equation, the optimum GF height for the present configuration is found to be 1.8%.

For the sake of clarity, the values of *C*_*L*_ and *C*_*D*_ for GF positions of only *S* = 0%, 4%, 10%, 15%, and 20% are presented. As GF moves upstream, *C*_*L*_ decreases slowly first and decreases rapidly when *S* > 10%.

Maximum *C*_*L*_ values compared with the airfoil without GF are increased by 31.4%, 29.0%, 25.0%, 15.8%, and 1.8% when flap at positions *S* = 0%, 4%, 10%, 15%, and 20% is used, respectively.

The lift enhancing capability clearly decreases as the GF is moved away from trailing edge. For *S* > 10%, maximum *C*_*L*_ values drop rapidly.

### 5.2. Drag Coefficient

*L/D* ratio increases with 1.5% GF height, but drag increases lowering* L/D* ratio if the flap is moved away from trailing edge. Drastic rise in drag for *S* > 10% results in lower* L/D* ratio. However, experimental results do not predict same trends. From experiments,* L/D* ratio always decreases independent of the position of the flap.

For a given* L/D* ratio, *C*_*L*_ values are higher for flaps at greater distance from trailing edge. Experimental drag values show a drastic rise in drag with GF placed at *S* = 4 to 6% for AoA from 4° to 8°. But these trends are not predicted computationally, where increment in *C*_*D*_ is almost continuous.

### 5.3. Static Pressure Distribution

**Figure 11:**
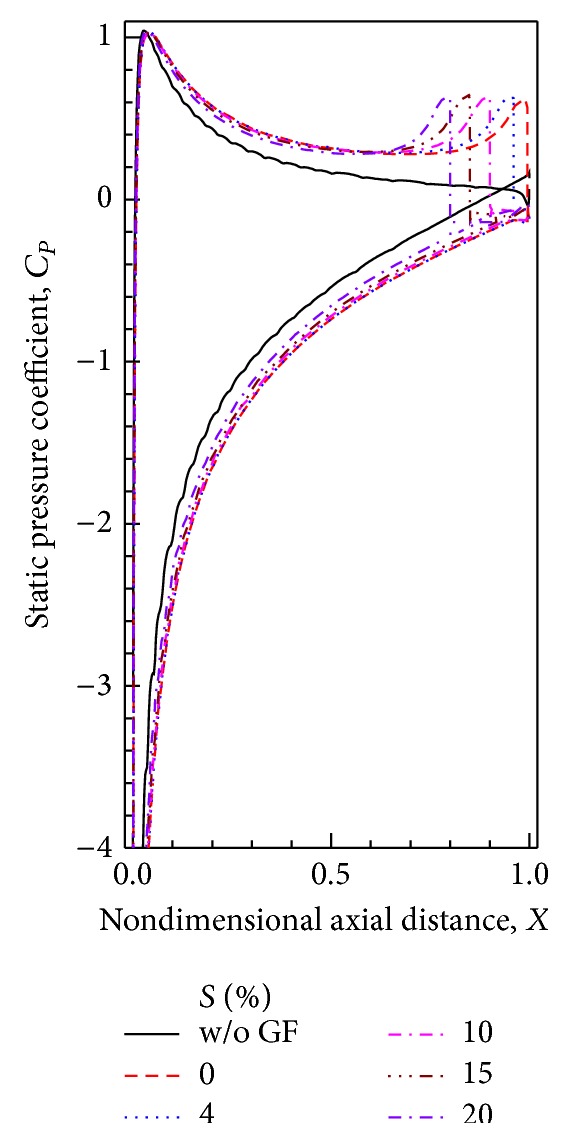
Static pressure distributions for different GF positions at AoA = 12°.

Distribution of static pressure coefficient on the airfoil surface obtained computationally with 1.5% height GF at AoA = 12° is shown in Figure 11. As the GF moves away from trailing edge, increment in suction decreases but at a very slow rate up to the position of *S* = 15%. Increase in pressure near the GF is almost the same for all the positions. However the static pressure on the pressure surface decreases immediately downstream of the flap reaching the value on the suction surface. Hence the lift coefficient is reduced due to less area with large pressure difference between suction and pressure surfaces with Gurney flap moved upstream.

### 5.4. Pathlines and Turbulence Intensities

**Figure 12:**
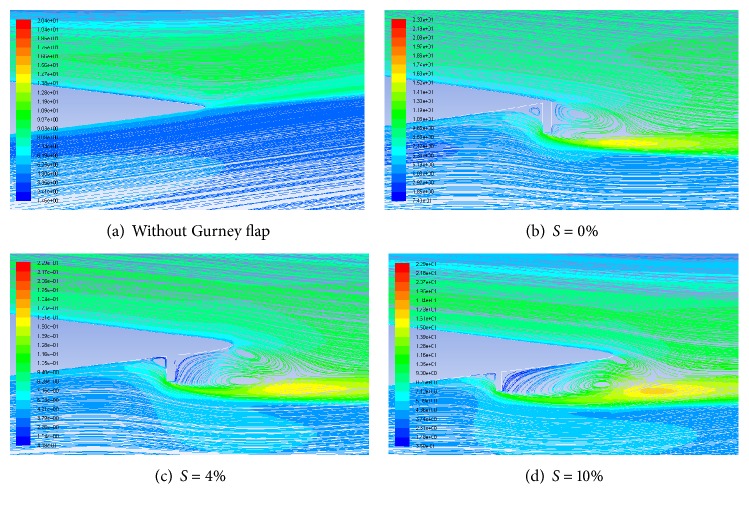
Pathlines and turbulence intensities for different positions with 1.5% height GF at AoA = 12°.

Flow near the trailing edge of airfoil without and with 1.5% height GF at different Gurney flap positions at an AoA of 12° is shown in Figure 12. The maximum turbulent intensity in these vortices increases as the Gurney flap is moved upstream. However, as the distance of the flap increases, the vortex near to suction surface is not formed completely. For *S* > 10%, vortex almost terminates which results in decreased suction and hence decrease in the lift enhancement.

## 6. Effect of Mounting Angle of GF on Airfoil Aerodynamics

### 6.1. Lift Coefficient

**Figure 13:**
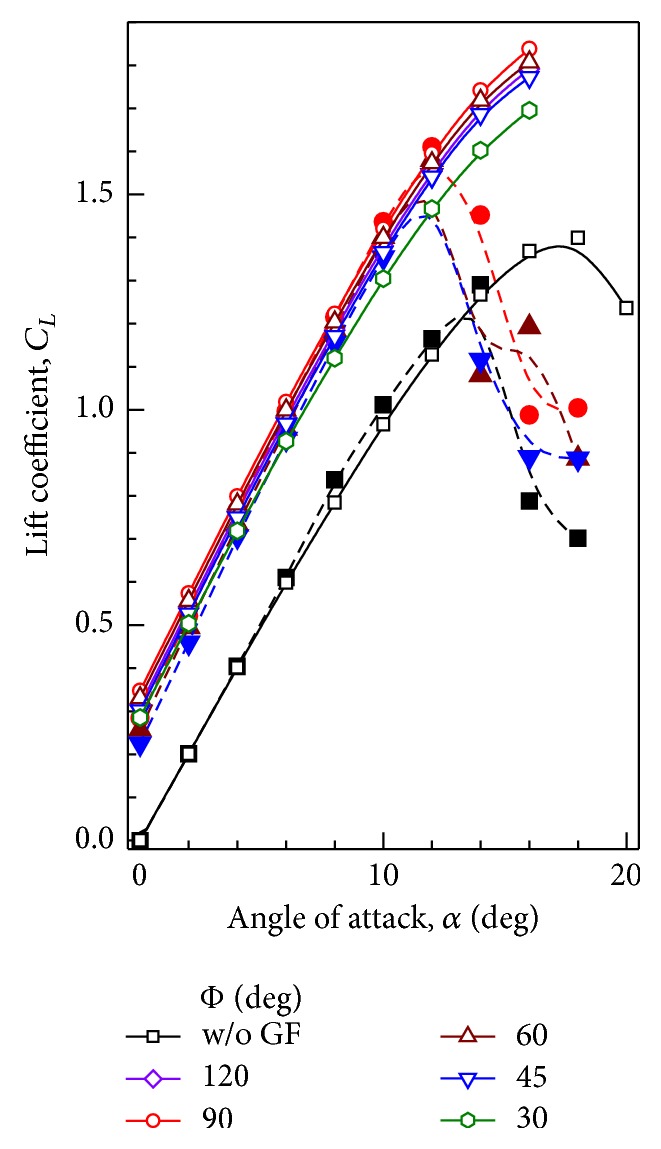
Variation of lift coefficient with angle of attack for GF mounting angles. Open symbols + solid line: Computational results. Closed symbols + dashed line: Experimental results [12].

**Figure 14:**
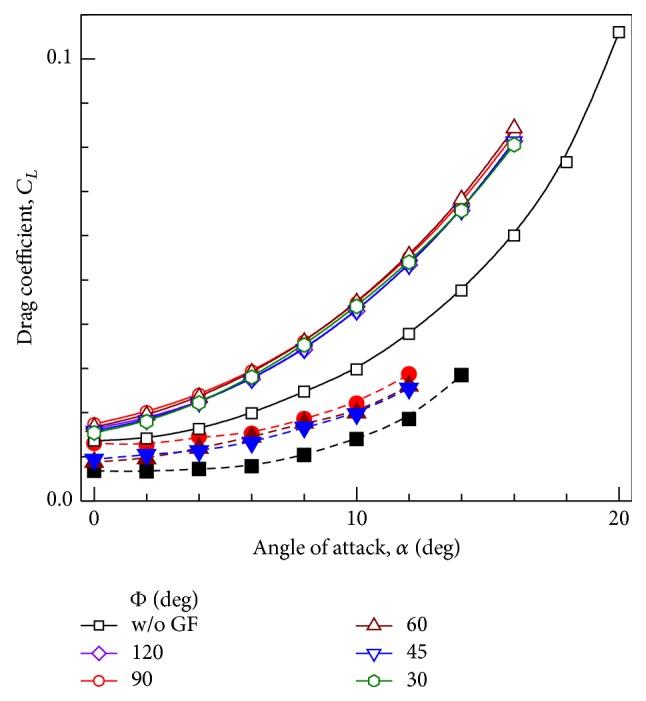
Variation of drag coefficient with angle of attack for GF mounting angles. Open symbols + solid line: Computational results. Closed symbols + dashed line: Experimental results [12].

Experimental and computational values of *C*_*L*_ and *C*_*D*_ for different mounting angles of GF are compared in Figures 13 and 14, respectively. For the sake of clarity, the values of *C*_*L*_ and *C*_*D*_ for GF mounting angles of only Φ = 30°, 45°, 60°, 90°, and 120° are presented. When compared with airfoil with no GF, installation of GF increases the maximum obtained *C*_*L*_ by 28.0%, 29.0%, 31.4%, 30.4%, 29.0%, 26.7%, and 21.0% when GF is mounted at Φ = 120°, 105°, 90°, 75°, 60°, 45°, and 30° angles, respectively. As long as the mounting angle is between 60° and 120°, lift coefficient does not vary on large scale. Similar observations are made through experimental results. Computed and experimental values of *C*_*L*_ at 12° angle of attack (near stall) for various mounting angles of GF are presented in Table 3. For Φ < 45°, *C*_*L*_ drops very rapidly thereby decreasing the lift enhancement of GF.

### 6.2. Drag Coefficient

For initial angles of attack,* L/D* ratio increases as the mounting angle decreases from perpendicular position with lowest* L/D* ratio for Φ = 90°. Also maximum* L/D* ratio is obtained for Φ = 45°. For higher angles of attack,* L/D* ratio for airfoil without GF decreases very rapidly, in contrast to lower rate of decrease in airfoil with GF. Similar observations are also made experimentally. For Φ < 45°,* L/D* ratio decreases due to steep decrease in *C*_*L*_ values.

### 6.3. Static Pressure Distribution

**Figure 15:**
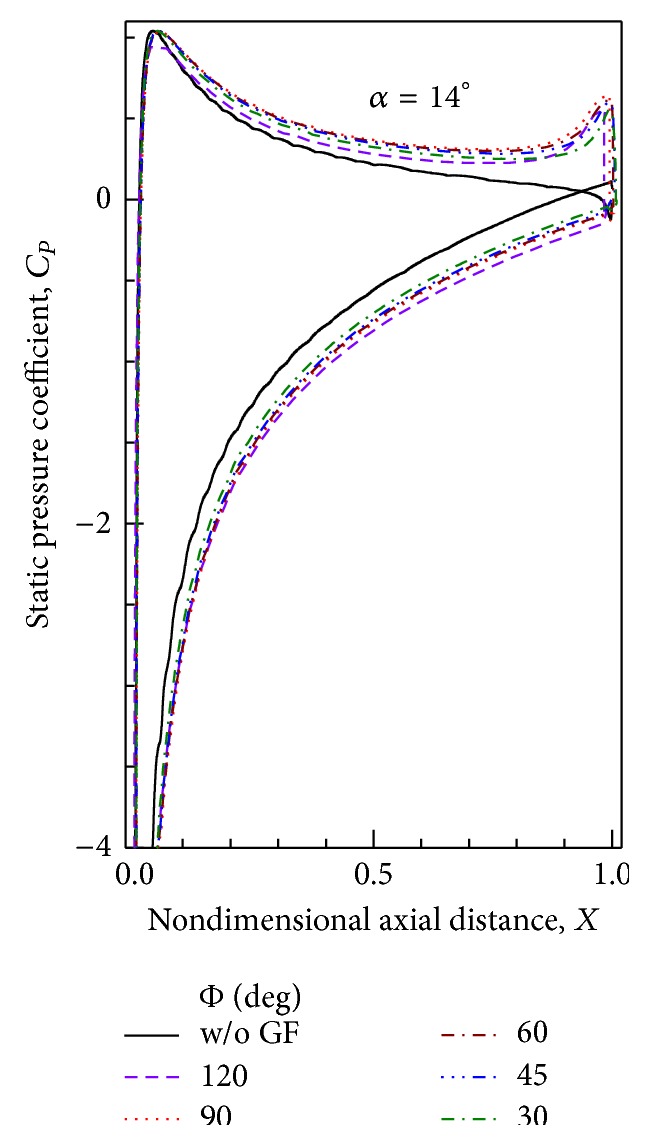
Static pressure distributions for different GF mounting angles at AoA = 14°.

Distribution of static pressure coefficient on the airfoil surface obtained computationally with 1.5% height GF at an AoA = 14° is shown in Figure 15. Mounting angle of Gurney flap seems to affect the static pressure distribution only near the trailing edge. For Φ = 30°, there is visible decrease in suction and pressure on respective airfoil surfaces. Also the location of rise in suction and pressure moves downstream as the mounting angle is decreased. This is because the Gurney flap mounted at angle Φ < 90° slightly increases the overall length of the airfoil beyond the actual trailing edge. The amount of maximum suction pressure is also almost the same for all the cases except for Φ = 30° for which maximum suction is ≈7% less than other tested mounting angles.

### 6.4. Pathlines and Turbulence Intensities

**Figure 16:**
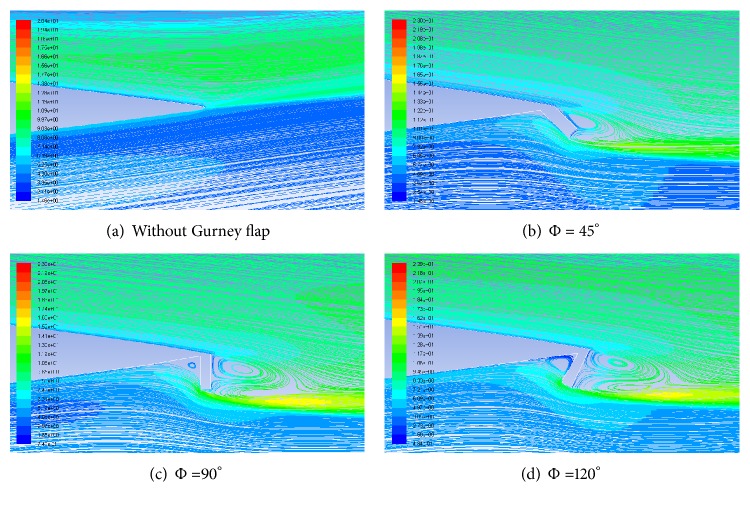
Pathlines and turbulence intensities for different mounting angles with 1.5% height GF at AoA = 12°.

Flow near the trailing edge of airfoil without and with 1.5% height GF at different Gurney flap mounting angles at an AoA of 12° is shown in Figure 16 via pathlines superimposed with contours of turbulence intensity. As the mounting angle of the flap decreases, the vortex near to suction surface is not formed completely. For Φ < 45°, vortex almost disappears which results in decreased suction and hence drastic decrease in the lift coefficient. The turbulence intensity at the airfoil exit increases with increasing mounting angle. The most probable reason for this may be due to increased strength of vortex due to strong obstruction in the path of flow.

## 7. Conclusions

From the present computational investigations, the following major conclusions are drawn.

(1) The agreement between computed and experimental values of lift coefficient is very good up to stall angle. Near and above stall angle, the lift coefficient continues to increase. An improved turbulence may provide better results near and above stall angle.
(2) The agreement between computed and experimental static pressure distribution on the airfoil surfaces is good even near GF.
(3) Lift enhancement is achieved for greater heights but at the expense of increased drag. The rate of lift increment decreases for greater heights and drag increases rapidly for *H* > 2%.
(4) Lift decreases when GF is moved upstream the trailing edge. Moving GF upstream decreases the effective area of pressure difference on the airfoil; hence GF should be positioned within 10% distance from the trailing edge.
(5) Decreasing the mounting angle decreases the drag but lift is also decreased.* L/D* ratio is found to be maximized at Φ = 45°. Hence GF should always be mounted at Φ > 45° to prevent major lift loss.

## Figures and Tables

**Table 1:** Comparison of *C*_*L*_ values at AoA = 12° for different GF heights.

*H*	Computational *C*_*L*_	% increase	Experimental *C*_*L*_	% increase
0.0	1.128	—	1.165	—
0.5	1.382	22.6	1.299	11.5
1.0	1.497	8.3	1.446	11.3
1.5	1.595	6.6	1.528	5.6
2.0	1.667	4.5	1.580	3.5
3.0	1.782	6.9	1.646	4.1
4.0	1.877	5.4	—	—

**Table 2:** Comparison of *C*_*L*_ values for different positions of 1.5% GF at AoA = 12°.

*S* (%)	Computational *C*_*L*_	% decrease	Experimental *C*_*L*_	% decrease
0.0	1.595	—	1.610	—
2.0	1.598	−0.2	1.610	0.0
4.0	1.588	0.6	1.602	0.5
6.0	1.569	1.2	1.560	2.6
10.0	1.531	2.4	—	—
15.0	1.478	3.5	—	—
20.0	1.374	7.0	—	—

**Table 3:** Comparison of *C*_*L*_ values for different mounting angles of 1.5% GF at AoA =12°.

Φ (Deg.)	Computational *C*_*L*_	% decrease in *C*_*L*_ with respect to Φ = 90°	Experimental *C*_*L*_	% decrease in *C*_*L*_ with respect to Φ = 90°
120	1.554	2.61	—	—
105	1.568	1.73	—	—
90	1.595	—	1.611	—
75	1.592	0.23	—	—
60	1.573	1.39	1.580	1.87
45	1.541	3.39	1.544	4.10
30	1.467	8.00	—	—

## Nomenclature

AoA, *α*: Angle of attack (Deg.)
*C*_*D*_: Drag coefficient
CFD: Computational fluid dynamics
Ch: Airfoil chord (m)
*C*_*L*_: Lift coefficient
*C*_*P*_: Static pressure coefficient
Expt.: Experimental
FMG: Full multigrid
GF: Gurney flap
*H*: GF height in percentage of chord length
*L/D*: Lift to drag ratio
RANS: Reynolds averaged Navier stokes
Re: Reynolds number = V Ch/*ν*
RNG: Renormalization group
*S*: Distance in percentage of chord length from trailing edge
TKE: Turbulent kinetic energy
*U*: Free stream velocity (m/s)
*ε*: Rate of dissipation of turbulent kinetic energy (m^2^/s^3^)
Φ: Mounting angle with the axial chord (Deg.)
*k*: Turbulent kinetic energy (m^2^/s^2^)
*ν*: Kinematic viscosity (m^2^/s).
